# M1/M2-macrophage phenotypes regulate renal calcium oxalate crystal development

**DOI:** 10.1038/srep35167

**Published:** 2016-10-12

**Authors:** Kazumi Taguchi, Atsushi Okada, Shuzo Hamamoto, Rei Unno, Yoshinobu Moritoki, Ryosuke Ando, Kentaro Mizuno, Keiichi Tozawa, Kenjiro Kohri, Takahiro Yasui

**Affiliations:** 1 Department of Nephro-urology, Nagoya City University Graduate School of Medical Sciences, Nagoya, Japan

## Abstract

In our previous report, M2-macrophage (Mφs) deficient mice showed increased renal calcium oxalate (CaOx) crystal formation; however, the role of Mφs-related-cytokines and chemokines that affect kidney stone formation remains unknown. Here, we investigated the role of M1/M2s in crystal development by using *in vitro* and *in vivo* approaches. The crystal phagocytic rate of bone marrow-derived M2Mφs was higher than that of bone marrow-derived Mφs and M1Mφs and increased on co-culture with renal tubular cells (RTCs). However, the amount of crystal attachment on RTCs reduced on co-culture with M2Mφs. In six hyperoxaluric C57BL/6J mice, M1Mφ transfusion and induction by LPS and IFN-γ facilitated renal crystal formation, whereas M2Mφ transfusion and induction by IL-4 and IL-13 suppressed renal crystal formation compared with the control. These M2Mφ treatments reduced the expression of crystal-related genes, such as osteopontin and CD44, whereas M1Mφ treatment increased the expression of pro-inflammatory and adhesion-related genes such as IL-6, inducible NOS, TNF-α, C3, and VCAM-1. The expression of M2Mφ-related genes was lower whereas that of M1Mφ-related genes was higher in papillary tissue of CaOx stone formers. Overall, our results suggest that renal crystal development is facilitated by M1Mφs, but suppressed by M2Mφs.

Kidney stone disease has markedly increased both in adult and pediatric populations^1^,^2^,^3^. Kidney stones have epidemiological as well as histopathological associations with kidney disease and may lead to chronic kidney disease and end-stage renal disease^4^; however, the exact mechanism of renal crystal formation remains unknown. Our research group has focused on the therapeutic role of renal mononuclear phagocytes, especially of macrophages (Mφs), in the regulation of crystal development using *in vitro* and *in vivo* approaches^5^,^6^,^7^,^8^. Our previous studies showed spontaneous disappearance of renal calcium oxalate (CaOx) crystals in hyperoxaluric mice with the expression of various Mφ-related cytokines and chemokines. Other related studies reported that urinary and renal tubular CaOx monohydrate (COM) crystals are broken down and dissolved in the presence of Mφs, whereas Mφ migration occurs concurrently with the crystal formation prior to the disappearance^9^,^10^. An *in vitro* study also demonstrated that the co-culture of renal tubular cells (RTCs) and RAW 264.7, a murine Mφ cell line, facilitates the adherence of COM crystals on RTCs via the expression of pro-inflammatory adipocytokines such as monocyte chemoattractant protein 1 (MCP-1), interleukin-6 (IL-6), and tumor necrosis factor (TNF)-α. Mφs have two major poles, one classically activated (M1) and another alternatively activated (M2), which are probably associated with crystal formation or disappearance and need to be considered as two opposite Mφ types^11^,^12^.

The population of renal mononuclear phagocytes, including Mφs, has diverse reactions in kidney disease^13^,^14^,^15^. Several reports have demonstrated that M2-like Mφs have anti-inflammatory and tissue healing effects on the *in vivo* models of nephropathy and ischemia/reperfusion acute kidney injury^16^,^17^,^18^,^19^. However, pro-inflammatory M1-like Mφs worsen the renal condition, leading to chronic kidney disease and fibrosis^20^,^21^. Additionally, our most recent study demonstrated that a substantial number of M1-like Mφs contributed to the development of renal crystal deposits in metabolic-syndrome model mice^22^. We also found that colony stimulating factor (CSF)-1 signaling suppressed renal crystal formation by the induction of M2-like Mφs in M2-deficient mice, revealing the potential therapeutic role of M2s and the differentiation of M1s^23^.

In this study, we investigated the role of M1Mφs and M2Mφs in renal CaOx crystal development using *ex vivo* induction of bone-derived Mφs (BMMs) with both *in vitro* and *in vivo* approaches. *Ex vivo* M2Mφs showed significant ability of COM crystal phagocytosis and anti-adherence on RTCs. The adoptive transfusion and selective induction by M2Mφs attenuated renal crystal formation, whereas those by M1Mφs facilitated renal crystal formation. We further discovered the gene expression profile of human renal papillae related to Mφs, and showed that CaOx stone formers had lower expression of M2Mφ-related genes than the controls.

## Results

### M2Mφs reduce COM crystal attachment to renal tubular cells *in vitro*

**Figure 1:**
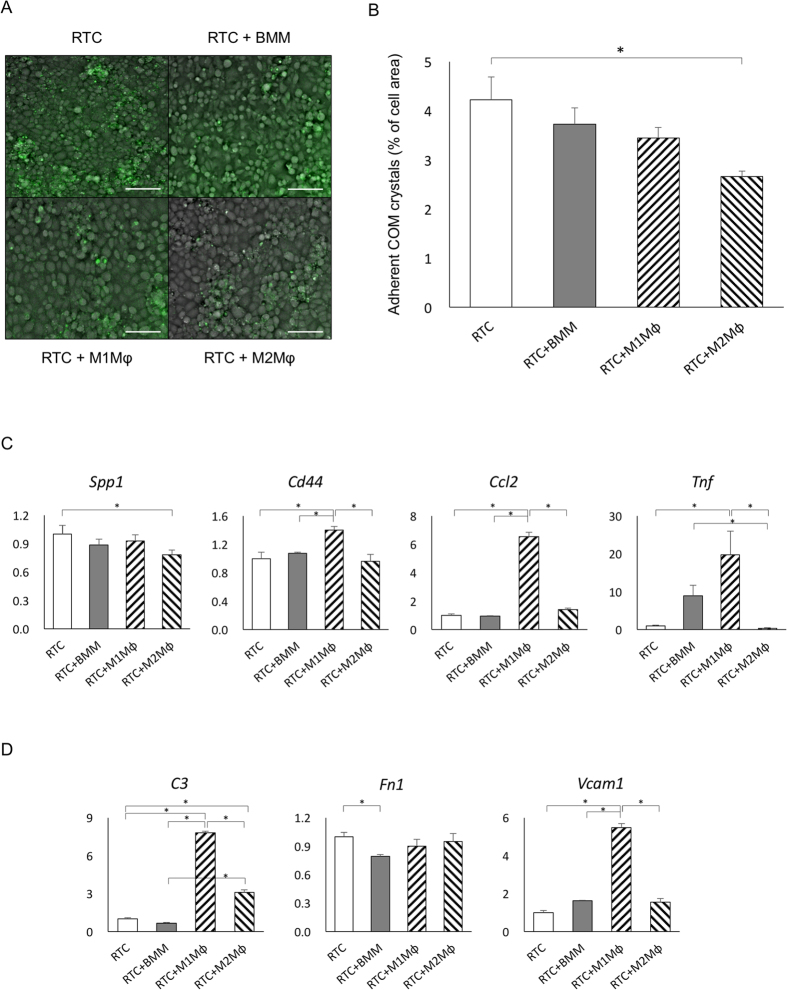
Analysis of the role of M1 and M2 macrophages in the adherence of calcium oxalate (CaOx) monohydrate (COM) crystals on renal tubular cells *in vitro*. (**A**) Representative fluorescent micrographs show the attachment of fluorescein isothiocyanate (FITC) -labeled COM crystals on renal tubular cells. Scale bar = 50 μm. (**B**) Area occupied by crystalline material was measured and expressed as percent of image area. Asterisk indicates *p* < 0.05. (**C**) Expression of crystal- and inflammation-related genes. (**D**) Expression of adherence-related genes. The expression of each gene was determined by quantitative reverse transcription polymerase chain reaction (qRT-PCR) using TaqMan assays. Control values are the average of the data for renal tubular cells (RTCs). Data are presented as means ± standard errors. N = 6 for each group. Asterisk indicates significant differences at *p* < 0.05. *Spp1*, secreted phosphoprotein 1; *Ccl2*, chemokine (C-C motif) ligand 2; *Tnf*, tumor necrosis factor; *Fn1*, fibronectin 1; *Vcam1*, vascular cell adhesion molecule 1.

The amount of adherent fluorescent-labeled COM crystals (Fig. 1A) was considerably lower in the RTC+M2Mφ group than in the RTC group, but no significant differences were identified among the RTC groups co-cultured with different type Mφs (Fig. 1B).

The expression of secreted phosphoprotein 1 (*Spp1*, encoding osteopontin [OPN]) in the RTC+M2Mφ group was lower than that in the RTC group, whereas the expression of *Cd44* and chemokine (CC motif) ligand 2 (*Ccl2*, encoding MCP-1) was higher in the RTC+M1Mφ group than in the other groups. The expression of tumor necrosis factor (*Tnf*, encoding TNFα) in the RTC+M1Mφ group was the highest, whereas that in the RTC+M2φ group was the lowest among the four groups (Fig. 1C). The expression of complement component 3 (*C3*) and vascular cell adhesion molecule 1 (*Vcam1*) in the RTC+M1Mφ group was significantly higher than that in the other groups; however, no significant differences were identified in the expression of fibronectin 1 (*Fn1)* among groups (Fig. 1D).

### M2Mφs phagocytize COM crystals *in vitro*

**Figure 2:**
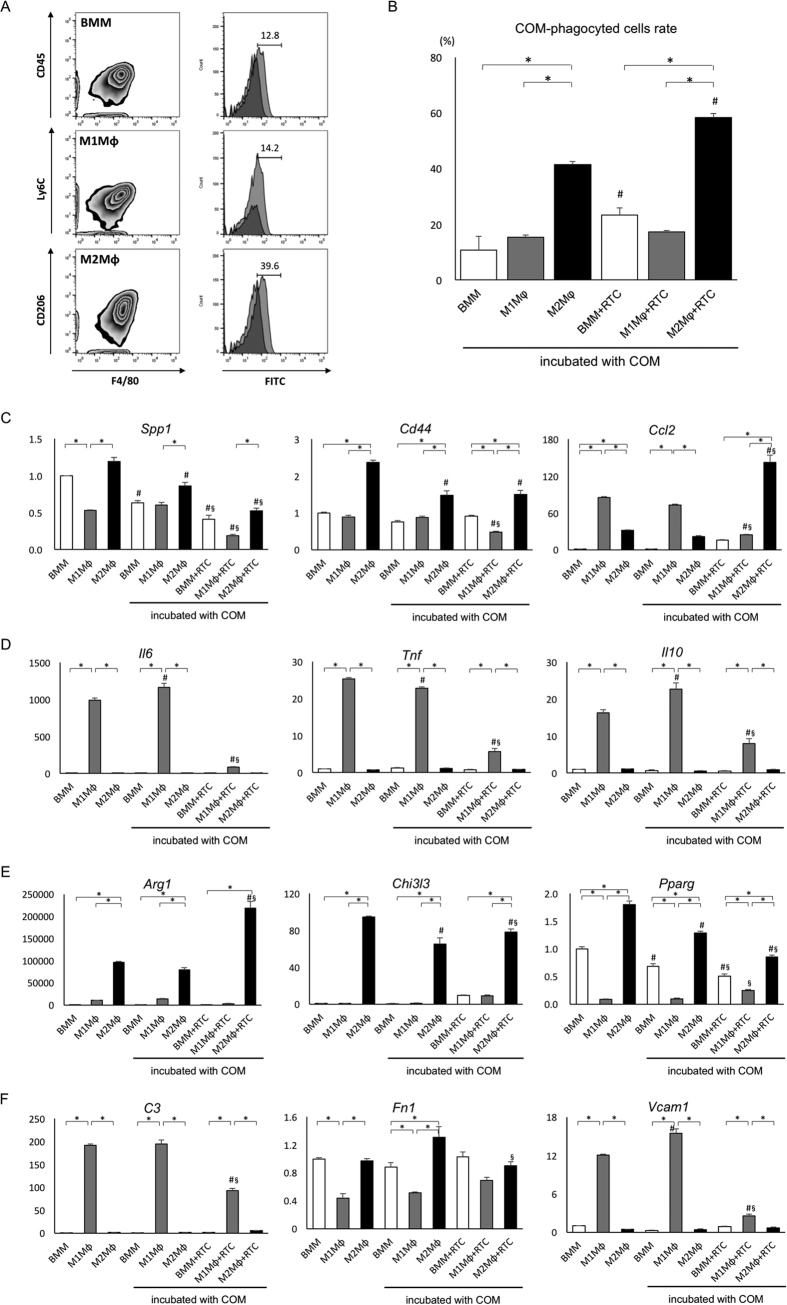
Analysis of the ability of M1 and M2 macrophages to phagocytize calcium oxalate (CaOx) monohydrate (COM) crystals *in vitro*. (**A**) Schematic representation using flow cytometry. Detection of bone marrow-derived macrophages (upper), M1 macrophages (middle), and M2 macrophages (bottom) with fluorescein isothiocyanate (FITC) -labeled COMs phagocytized by each macrophage type (right). (**B**) Quantitative analysis of phagocytic macrophages. The ratios of COM phagocytic macrophages were evaluated by flow cytometry. Data are presented as means ± standard errors. N = 6 for each group. *Indicates significant differences among three different macrophages at *p* < 0.05; ^#^indicates significant differences in the mono-culture of each macrophage group at *p* < 0.05. (**C**) Expression of crystal-related genes. (**D**) Expression of M1 macrophage-related genes. (**E**) Expression of M2 macrophage-related genes. (**F**) Expression of adherence-related genes. The expression of each gene was determined by quantitative reverse transcription polymerase chain reaction (qRT-PCR) using TaqMan assays. Control values are the average of the data for bone marrow-derived Mφs (BMMs) without COM incubation group. Data are presented as means ± standard errors. N = 6 for each group. *Indicates significant differences among three different macrophages at *p* < 0.05; ^#^indicates significant differences in the mono-culture of each macrophage group at *p* < 0.05; ^§^indicates significant differences in the mono-culture of each macrophage group incubated with COM crystals at *p* < 0.05. *Spp1*, secreted phosphoprotein 1; *Ccl2*, chemokine (C-C motif) ligand 2; *Il6*, interleukin 6; *Tnf*, tumor necrosis factor; *Il10*, interleukin 10; *Arg1*, arginase 1; *Chi3l3*, chitinase 3-like 3; *Pparg*, peroxisome proliferator activated receptor gamma; *Fn1*, fibronectin 1; *Vcam1*, vascular cell adhesion molecule 1; *RTC*, renal tubular cell.

BMM-derived M1Mφs and M2Mφs (Fig. 2A) were detected by flow cytometry with staining for F4/80^+^Ly6C^+^ and F4/80^+^CD206^+^, respectively. M2Mφs had a higher phagocytic rate of COM crystals than BMMs or M1Mφs. The phagocytic ability of BMMs and M2Mφs increased on co-culture with RTCs (Fig. 2B).

The expression of *Spp1* and *Cd44* in M2Mφ mono-culture with or without COM incubation and in M2Mφs co-cultured with RTCs incubated with COM as well as the expression of *Ccl2* in the latter was markedly higher than that in the BMM and M1Mφ groups. The incubation with COM or co-culture with RTCs decreased the expression of *Spp1* and *Cd44*. The expression of *Ccl2* in the M1Mφ group with or without COM was higher than that in the BMM and M2Mφ groups (Fig. 2C). The expression of interleukin 6 (*IL6*), *Tnf*, and interleukin 10 (*IL10*) was considerably higher in the M1Mφ group than in the BMM and M2Mφ groups, and increased by COM exposure, whereas it decreased on co-culture with RTCs (Fig. 2D). Enzyme-linked immunosorbent assay (ELISA) of each Mφ culture supernatant showed that the expression of OPN, MCP-1, IL-6, and TNFα was the highest in the M1Mφ group. COM exposure decreased the secretion of OPN from each Mφ culture. Co-culture with RTCs decreased the expression of TNFα in the M1Mφ group and increased the expression of MCP-1 in the M2Mφ group and OPN in all Mφ groups (Supplementary Figure 1).

The expression of arginase (*Arg1*), chitinase 3-like 3 (*Chi3l3*, encoding Ym-1), and peroxisome proliferator activated receptor gamma (*Pparγ*) was considerably higher in the M2Mφ group than in the BMM and M1Mφ groups. The expression of *Arg1* and *Chi3l3* in the M2Mφ group increased on co-culture with RTCs. The expression of *Pparg* in the M1Mφ group was the lowest among the three Mφ groups (Fig. 2E).

The expression of *C3* and *Vcam1* was significantly higher, whereas the expression of *Vcam1* was lower in the M1Mφ group than in the BMM and M2Mφ groups. The expression of *C3* and *Vcam1* decreased on co-culture with RTCs (Fig. 2F).

### Renal and urinary CaOx crystals *in vivo*

**Figure 3:**
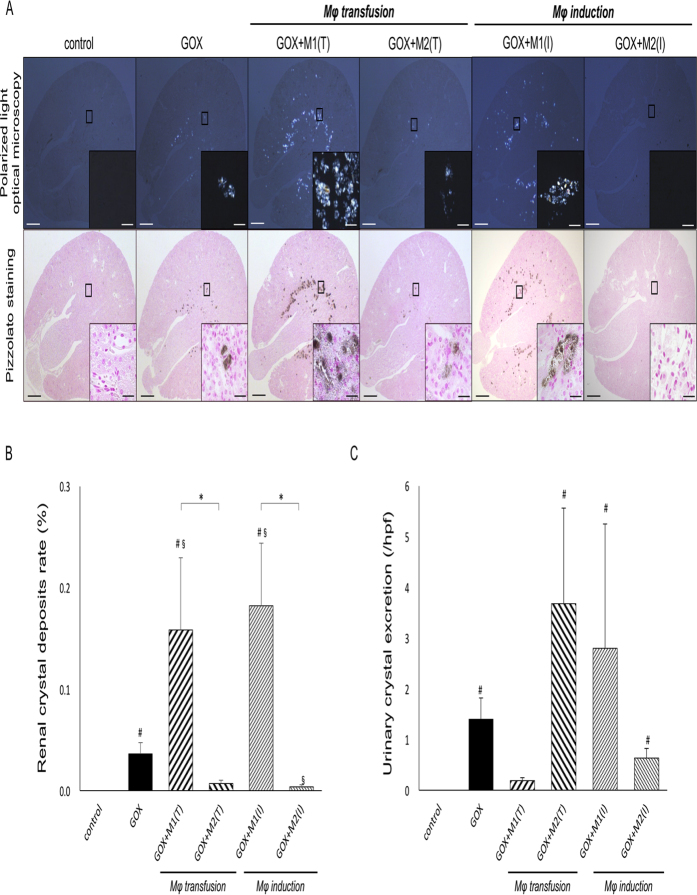
Morphological and quantitative distribution of renal calcium oxalate crystals. (**A**) Morphological distribution of renal calcium oxalate (CaOx) crystal deposits detected using polarized light optical microscopy and Pizzolato staining. Representative micrographs obtained at d 6. Scale bar = 500 μm; inset scale bar = 25 μm. (**B**) The ratio of areas with renal crystal deposition. Crystallization in each kidney section was quantified by calculating the ratio (%) of the area containing crystals to the entire kidney section using Image Pro Plus. (**C**) The number of urinary crystals per high-powered field. At d 6, 24-h urine samples were collected in a metabolic cage, and CaOx crystals were counted at 400× magnification. N = 6 for each group. Data are presented as means ± standard errors. *Indicates significant differences between the M1 and M2Mφ groups in the same treatment at *p* < 0.05; ^#^indicates significant differences compared with the control group at *p* < 0.05; ^§^indicates significant differences compared with the glyoxylate (GOX)-treated group. M1 (T) and M2 (T) indicate transfusion, whereas M1 (I) and M2 (I) induction treatment of each macrophage.

CaOx crystals were identified in the renal tubular lumens at the corticomedullary region in glyoxylate (GOX)-treated mice under the polarized light optical microscope with Pizzolato staining (Fig. 3A).

The amount of renal crystals was significantly higher in the GOX+M1 (Transfusion; T) and GOX+M1 (Induction; I by daily i. p. administration of LPS and IFN-γ) groups than in the other groups (*p* < 0.03). The amount of renal crystals in the GOX+M2 (I by daily i. p. administration of IL-4 and IL-13) group was lower than that in the GOX group (Fig. 3B). The amount of crystalluria was higher in the GOX-treated groups, except for the GOX+M1 (I) group; however, no significant differences were identified in Mφ transfusion or induction between M1Mφs and M2Mφs (Fig. 3C). The ratios of renal deposited/urinary excreted crystals were evaluated to compare the crystal clearance from the kidney to the renal collecting system among the groups, and the GOX+M1 (T) group showed a higher ratio, whereas the GOX+M2 (T) and (I) groups had lower ratios compared to the GOX group (Supplementary Figure 2).

### Serum and urinary variables *in vivo*

Serum creatinine and phosphorus levels in the GOX+M1 (I) group were significantly higher than those in the control, GOX, and GOX (I) groups (p < 0.05). Serum sodium levels in the GOX+M1 (I) and M2 (I) groups were higher than those in the control group.

Urinary volume in the GOX and GOX+M2 (I) groups was higher than that in the control groups. All GOX-treated groups had considerably higher levels of urinary oxalate compared to the control group. There were no significant differences in the other serum and urinary variables among the 6 experiment groups (Table 1).

### M1Mφs facilitate whereas M2Mφs suppress the expression of crystal-related and adherence-related genes *in vivo*

**Figure 4:**
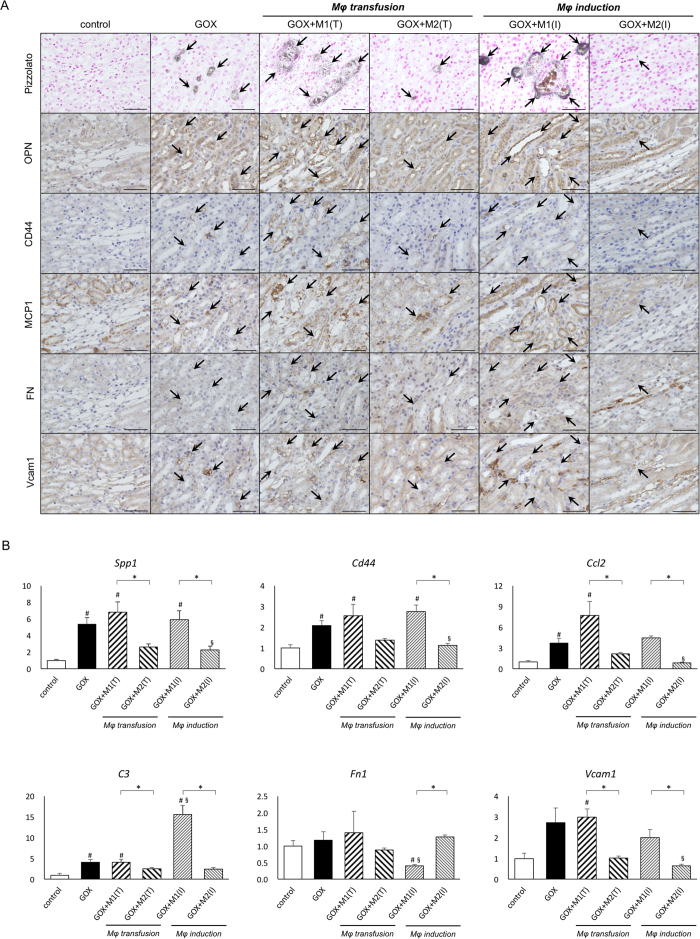
Evaluation of crystal-related and adherence-related gene expressions. (**A**) Immunohistochemical distribution of gene expression in mouse kidneys harvested at d 6. Pizzolato staining indicated calcium oxalate (CaOx) crystal deposits (arrows). OPN, osteopontin; MCP-1, monocyte chemoattractant protein 1; FN, fibronectin; Vcam1, vascular cell adhesion molecule 1. Scale bar = 50 μm. (**B**) The expression of genes was determined by quantitative reverse transcription polymerase chain reaction (qRT-PCR) using TaqMan assays. Control values represent the average of the data for the control group. Data are presented as means ± standard errors. N = 6 for each group. *Indicates significant differences between the M1 and M2Mφ groups in the same treatment at *p* < 0.05; ^#^indicates significant differences compared with the control group at *p* < 0.05; ^§^indicates significant differences compared with the glyoxylate (GOX)-treated group. *Spp1*, secreted phosphoprotein 1; *Ccl2*, chemokine (C-C motif) ligand 2; *Fn1*, fibronectin 1; *Vcam1*, vascular cell adhesion molecule 1. M1(T) and M2(T) indicate transfusion, whereas M1(I) and M2(I) indicate induction treatment of each macrophage.

OPN, CD44, and MCP-1 were detected in the renal tubular cells around the crystals, whereas FN and Vcam1 in the interstitial spaces of the renal tubular cells. The staining intensity of OPN, CD44, MCP-1, and Vcam1 was stronger in the GOX+M1 (T) and GOX+M1 (I) groups, whereas that of FN was stronger in the GOX+M2 (T) group than in other groups (Fig. 4A).

Quantitative reverse transcription polymerase chain reaction (qRT-PCR) revealed a marked increase in the expression of *Spp1*, *Cd44*, *Ccl2*, and *C3* in the GOX group. In the M1Mφ-treated groups, the expression of *Spp1*, *Cd44*, *Ccl2*, *C3*, and *Vcam1* was considerably higher than that in the M2Mφ-treated groups. The expression of *Fn1* was lower in the GOX+M1 (I) group, but higher in the GOX+M2 (I) group compared with the control and GOX groups (Fig. 4B).

### CaOx crystal development is related to the increase of M1-like Mφs that is suppressed by M2 treatment *in vivo*

**Figure 5:**
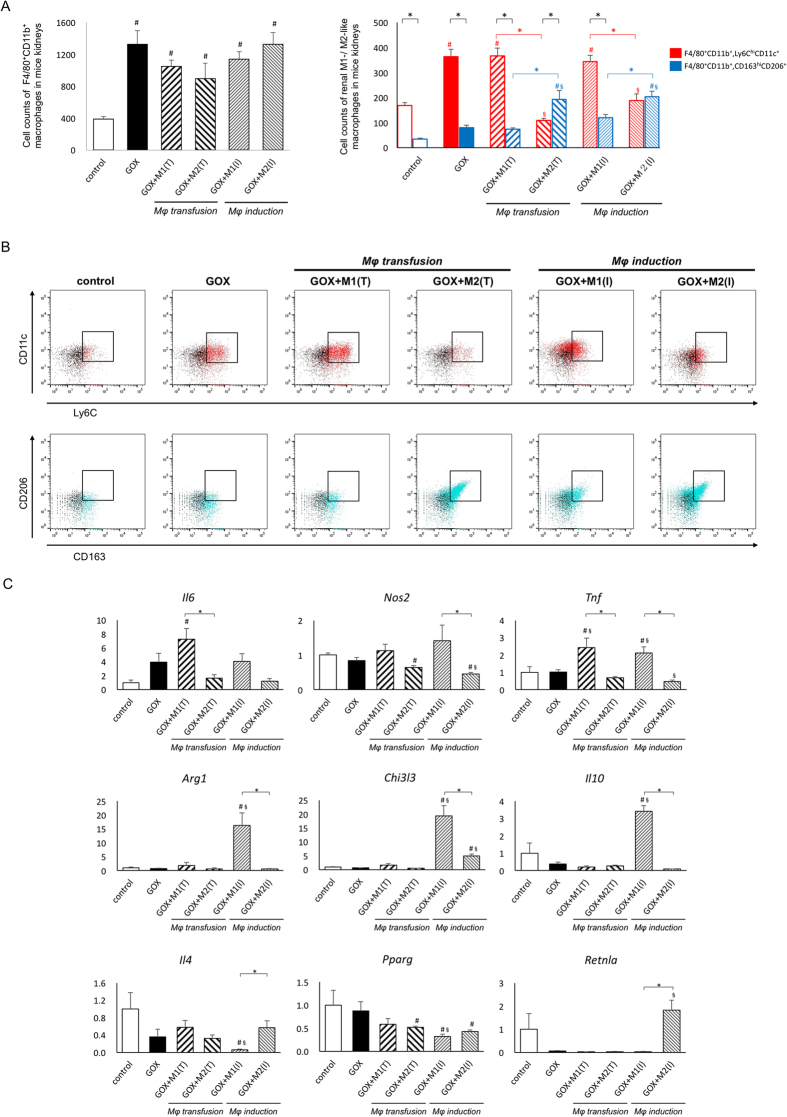
Evaluation of renal macrophages and their related gene expression. (**A**) Representative bar graphs demonstrate the number of renal F4/80^+^CD11b^+^ macrophages (left panel) and quantification of F4/80^+^CD11b^+^ Ly6C^hi^CD11c^+^M1-like macrophages (red bar) and F4/80^+^CD11b^+^CD163^hi^CD206^+^M2-like macrophages (blue bar) (right panel). Data are presented as means ± standard errors. N = 4 for each group. *Indicates significant differences between the M1 and M2-like macrophages in the same group (black font) and between M1 and M2 treatment groups (either red or blue font) at *p* < 0.05; ^#^indicates significant differences compared with the control group at *p* < 0.05; ^§^indicates significant differences compared with the glyoxylate (GOX)-treated group. The gating strategy for F4/80^+^CD11b^+^ cells as pan, F4/80^+^CD11b^+^Ly6C^hi^CD11c^+^ cells as M1-like and F4/80^+^CD11b^+^CD163^hi^CD206^+^ cells as M2-like macrophages was described in our previous study^23^. (**B**) Representative flow cytometry plots of F4/80^+^CD11b^+^Ly6C^hi^CD11c^+^ or F4/80^+^CD11b^+^CD163^hi^CD206^+^ macrophages are shown. (**C**) The expression of genes was determined by quantitative reverse transcription polymerase chain reaction (qRT-PCR) using TaqMan assays. Control values represent the average of the data for the control group. Data are presented as means ± standard errors. N = 6 for each group. *Indicates significant differences between the M1 and M2Mφ groups in the same treatment at *p* < 0.05; ^#^indicates significant differences compared with the control group at *p* < 0.05; ^§^indicates significant differences compared with the glyoxylate (GOX)-treated group. *Il6*, interleukin 6; *Nos2*, inducible nitric oxide synthase 2; *Tnf*, tumor necrosis factor; *Arg1*, arginase 1; *Chi3l3*, chitinase 3-like 3; *Il10*, interleukin 10; *Il4*, interleukin 4; *Pparg*, peroxisome proliferator activated receptor gamma; *Retnla*, resistin like alpha. M1(T) and M2(T) indicate transfusion, whereas M1(I) and M2(I) induction treatment of each macrophage.

Flow cytometry demonstrated that the number of renal pan Mφs, detected as F4/80^+^CD11b^+^ cells, significantly increased in all the GOX-treated groups (*p* < 0.01); however, no significant differences were identified among them (Fig. 5A left). Additionally, the number of renal M1-like Mφs, detected as F4/80^+^CD11b^+^Ly6C^hi^CD11c^+^ cells, was significantly higher than that of M2-like Mφs, detected as F4/80^+^CD11b^+^CD163^hi^CD206^+^cells, in the control, GOX, GOX+M1 (T), and GOX+M1 (I) groups. The number of M1-like Mφs was also higher in the GOX, GOX+M1 (T), and GOX+M1 (I) groups than in the control group. However, the number of M2-like Mφs was higher, whereas that of M1-like Mφs was lower in the GOX+M2 (T) and GOX+M2 (I) groups compared with the GOX-treated and M1-treated groups (Fig. 5A right and 5B).

The expression of *Il6* and *Tnf* was considerably higher in the GOX+M1 (T) group than in the control and GOX+M2 (T) groups. The expression of *Tnf*, *Arg1*, *Chi3l3*, and *Il10* was considerably higher in the GOX+M1 (I) group than in the control, GOX, and GOX+M2 (I) groups. The expression of interleukin 4 (*IL4*) and *Pparg* was lower in the GOX+M1(I) group than in the control and GOX groups. The expression of *IL4* and resistin like alpha (*Retnla* found in inflammatory zone [FIZZ]1) was significantly higher in the GOX+M2 (I) group than in the GOX+M1 (I) group (Fig. 5C).

### Gene expression profiling of crystal-related, M1-related, and M2-related genes in human renal papillary tissues

The gene expression profiles of renal papillary tissues from CaOx stone formers were characterized, and no significant differences were identified in physical, serum, urinary backgrounds between CaOx stone formers and control patients (Supplementary Table S1).

**Figure 6:**
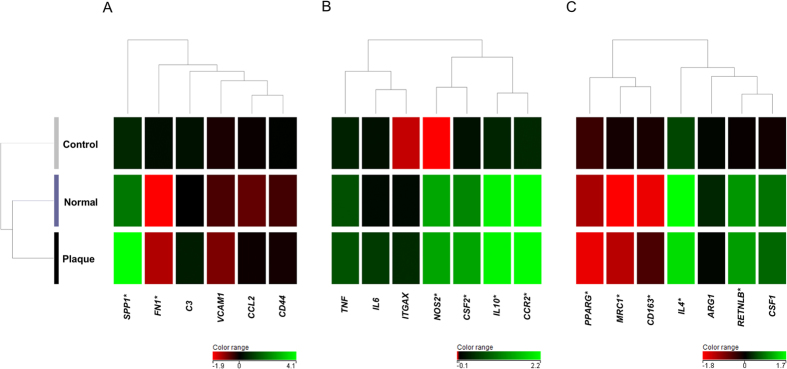
Microarray analysis of renal papillary tissue from calcium oxalate (CaOx) stone formers and control patients. Normal papillary tissues of non-stone patient were used as the control group. Normal papillary tissues and papillary tissues with Randall’s plaque of CaOx stone formers were used as normal and plaque groups, respectively. Cluster analysis of genes was performed among the three groups. Statistical comparisons of gene expression signals were implemented between the control and normal/plaque groups. *Indicates significant differences at *p* < 0.01 or >2-fold changes. (**A**) Crystal-related genes. (**B**) M1Mφ-related genes. (**C**) M2φ-related genes.

Cluster analysis separated the control group from the normal and plaque groups, results that were consistent with CaOx stone development. Among the crystal-related genes, the expression of *SPP1* was higher, whereas that of *FN1* was lower in the normal and plaque groups compared with the control group (Fig. 6A). The expression of M1-related genes, such as *NOS2*, *CSF2*, *IL10,* and CC chemokine receptor 2 (*CCR2*), was higher in the normal and plaque groups compared with that in the control group (Fig. 6B). However, the expression of *PPARG*, mannose receptor C type 1 (*MRC1*), and *CD163* was lower, whereas that of *IL4* and *RETNLB* was higher in the normal and plaque groups compared with that in the control group (Fig. 6C).

## Discussion

Since de Water *et al.* first reported encapsulation of interstitial crystals by macrophages and multinucleated giant cells^24^,^25^, several studies have investigated associations between renal crystal development and Mφ expression using *in vivo* and human sample approaches^7^,^8^,^9^,^26^,^27^. Because nephrolithiasis has several molecular, biological, and clinical similarities with atherosclerosis, its development mechanism, including the involvement of different Mφ types, can be predicted^22^,^28^,^29^,^30^. Using an *in vitro* approach, the present study showed that M2Mφs had significant ability of COM crystal phagocytosis and anti-adherence on RTCs. The expression of crystal-related and adherence-related genes, except for *Spp1* and *Fn1*, was increased by co-culturing the RTCs with M1Mφs; however, co-culture with M1Mφs did not affect the adherence rate of COM crystals on RTCs. The COM crystal phagocytic ability of M2Mφs was accelerated by co-culturing with RTCs, because it decreased the expression of pro-inflammatory genes, including *Il6, Tnf, Il10, C3*, and *Vcam1*, at both transcriptional and protein levels. Comparison of the amount of crystals deposited renally and excreted in the urine brought us to the interesting hypothesis that Mφs play a role in renal crystal development. The ratio of renal/urinary crystals was substantially lower in groups that underwent transfusion and induction of M2Mφs, whereas it was higher in groups that underwent transfusion and induction of M1Mφs compared to the GOX-treated control. These findings demonstrate that M2Mφs are capable of not only phagocytosing crystals but also clearing the crystals via prevention of their attachment to the RTCs. We previously demonstrated^23^ that the suppression of crystal-related molecules increased the phagocytic ability of murine M2Mφs. Additionally, CSF1-induced M2-like human Mφs have greater ability of crystal phagocytosis compared with CSF2-induced M1-like Mφs^31^. Therefore, the human kidney probably plays a protective role against crystal development because of the crystal phagocytic and anti-adherent ability of M2Mφs.

In hyperoxaluric rodents^32^,^33^, renal crystallization refers to the deposit of intraluminal crystals and increased urine mineral supersaturation and renal tubular cell damage, whereas some idiopathic CaOx stone development occurs through hydroxyapatite formation, and the resulting stones are termed as Randall’s plaque (RP)^34^,^35^,^36^,^37^,^38^. The kidney-developed CaOx crystals express pro-inflammatory cytokines^39^; crystal-related molecules, including OPN^40^, CD44^41^, and MCP-1^26^; and adhesion-related genes such as *C3*, *FN*, and *Vcam1*^8^,^28^. In this study, the expression of crystal-related and adhesion-related genes, except for *FN*, was increased in hyperoxaluric mice that had massive renal crystal deposits. The expression of these genes in tubular cells and the interstitial space around the crystal deposits was increased by M1 treatment but decreased by M2 treatment. Based on the association between the expression pattern of these genes and the amount of renal crystals, it was concluded that M1Mφs positively induced crystal formation. However, expression of *FN* was related to M2 treatment, as suggested by the present and previous studies^42^. M2Mφs increased the expression of *FN* but decreased that of other genes to prevent renal crystal formation.

No significant differences were identified in serum and urinary variables, except for oxalate excretion, among the control, GOX, and M1/M2Mφ-transfusion groups. The M1Mφ-induction group had increased serum creatinine and phosphorus levels and reduced urinary volume. The induction of M1Mφs by lipopolysaccharides (LPS) and interferon (IFN)-γ might cause renal dysfunction via acute renal injury; however, no changes in urinary variables were identified, revealing that there is no association between renal dysfunction and increased crystal deposition.

Pan-Mφs, which appeared in mice kidneys, increased with GOX treatment. Mφs in the GOX and M1Mφ-treated groups were mostly M1Mφs, indicating that they were responsible for the development and formation of crystal deposits in the kidney. However, when the number of M2Mφs reached that of M1Mφs, the renal crystal formation was suppressed. M1Mφ treatment increased the expression of pro-inflammatory molecules with crystal formation. M2Mφ induction increased the expression of anti-inflammatory genes, such as *Il4*, *Chi3l3,* and *Retnla*, and decreased that of *Nos2* and *Tnf*; whereas M2Mφ-transfusion did not affect any of these genes. It is assumed that crystal formation was differentially suppressed by M2Mφ transfusion or induction; the induction of M2Mφs by interleukins affected parenchymal cells, increased anti-inflammatory molecules, and reduced pro-inflammatory molecules, whereas the transfusion of M2Mφs improved crystal phagocytic and anti-adherent ability. Although a previous study reported that bone marrow-derived M2Mφs were difficult to maintain in their state compared with spleen-derived M2Mφs^43^, M2Mφ transfusion in the present study successfully exerted their role in the hyperoxaluric mouse kidney because of the short duration of the experimental period.

RPs provide a base for the development of idiopathic CaOx kidney stones, which begins in the basement membranes of thin Henle’s loops with calcium deposits^44^,^45^. The association between RPs and CaOx stone development is known^46^,^47^; however, a better understanding of RP pathogenesis is necessary for the prevention of nephrolithiasis. Our results showed that CaOx stone formers and non-stone formers had differences in the expression of crystal-related and Mφ-related genes. The expression of five pro-inflammatory-related genes, including *SPP1*, and two anti-inflammatory-related genes was higher, whereas that of four anti-inflammatory-related genes, including *FN1*, was lower both in the normal and plaque groups compared with the control group. The upregulation of M1Mφ-related genes and downregulation of some M2Mφ-related genes contributed to the development of human nephrolithiasis and RPs. Therefore, if the M1Mφ-dominant environment in human renal papilla was consistent with the *in vitro* and *in vivo* results of the present study, differences in the role of Mφs might be responsible for the development of intraluminal crystals and RP-related nephrolithiasis.

This study has some limitations: (1) The experimental *in vitro* and *in vivo* models do not accurately mimic the environment of kidney stones in patients who mostly have idiopathic CaOx stones related to RPs. Since both models have acute injuries and thus, excessive inflammation, M1/M2Mφs may easily affect crystal formation under specific circumstances. (2) Some parts of the role of Mφs are still unclear and need to be investigated in further studies. For instance, optimized capture of Mφ phagocytosis based on specific molecule activation both *in vitro* and *in vivo* would be useful for further understanding of their exact role with regard to crystals. (3) The M1/M2 Mφ treatment for patients with kidney stones needs further research because of the short-term effectiveness of bone marrow-derived M2Mφs and limited information on M1/M2 induction. (4) A relative higher number of human papillary samples is necessary for investigating CaOx stone formers and obtain accurate results.

Here, we investigated the differential roles of M1/M2Mφs in the development of renal CaOx using *ex vivo* induction of BMMs with both *in vitro* and *in vivo* approaches. Our results showed that M2Mφs suppressed renal CaOx stone development by crystal phagocytosis, inhibited crystal attachment to renal tubular epithelial cells, and reduced the expression of pro-inflammatory genes. These findings supported the therapeutic possibility of targeted Mφ-phenotype shifting from M1 to M2.

## Methods

### Induction of M1Mφs and M2Mφs from bone marrow cell cultures

BMMs grown in lymphocytes and 10% L-conditioned medium were generated as described previously^48^. At d 7, adherent cells were harvested and seeded at a density of 1.0 × 10^5^ cells cm^−2^. For priming experiments, BMMs were stimulated for 20 h with 100 ng ml^−1^ of LPS (Sigma-Aldrich, St. Louis, MO) and 20 ng ml^−1^ of GM-CSF (R&D Systems, Minneapolis, MN) for M1Mφs or 50 ng ml^−1^ of IL-4 (R&D Systems) and 10 ng ml^−1^ of M-CSF (Miltenyi Biotec, Bergisch Gladbach, Germany) for M2Mφs^49^,^50^.

### Evaluation of COM crystal adhesion to RTCs influenced by M1Mφs and M2Mφs

Fluorescence-labeled COM crystals were prepared as described previously^51^. We used murine renal tubular epithelial cells (M-1; American Type Culture Collection, Manassas, VA) and M1Mφs and M2Mφs that were induced from BMMs as described above. Four groups were established; the RTC group comprised only M-1 cells, whereas the co-culture groups included RTCs co-cultured with BMMs (RTC+BMM group), RTCs co-cultured with M1Mφs (RTC+M1Mφ group), and RTCs co-cultured with M2Mφs (RTC+M2Mφ group). RTCs (1.0 × 10^5^ cells cm^−2^) were grown in six-well culture plates (Corning Inc., Corning, NY). The cells were maintained in a humidified incubator at 37 °C with 5% CO_2_ for 24 h and then treated with 25 μg cm^−2^ of fluorescence-labeled COM crystals. Co-cultures (0.2 × 10^5^ cells cm^−2^) were grown in the upper inserts of the transwell. Cultured RTCs were observed under a DMI4000 B phase contrast microscope (Leica, Wetzlar, Germany) at 6 h after COM treatment.

### Evaluation of COM crystal internalization by RTCs, M1Mφs, and M2Mφs

We divided each type of cell into nine groups. BMMs, M1Mφs, and M2Mφs without COM crystal exposure were used as control groups. The other six groups were incubated with COM crystals; three types of Mφs (1.0 × 10^5^ cells cm^−2^) were mono-cultured or co-cultured with RTCs (0.2 × 10^5^ cells cm^−2^) using transwell inserts as described above. Each group of cultured cells was harvested after 6 h of COM treatment and then used for flow cytometry and qRT-PCR.

### Animal procedures

All experimental procedures were performed in accordance to the NIH Guide for the Care and Use of Laboratory Animals and approved by the Animal Care and Use Committee of the Faculty of Medicine, Nagoya City University Graduate School of Medical Sciences.

Male C57BL/6J wild-type mice were purchased from Charles River Japan Inc. (Yokohama, Japan). The mice were fed standard chow (AIN-93M; Oriental Yeast Co., Tokyo, Japan) and had free access to water. Equal numbers of 8-week-old male mice were assigned to six groups (n = 6): control, GOX (treated with glyoxylate), GOX+M1(T) (treated by glyoxylate and M1Mφ transfusion), GOX+M2(T) (treated by glyoxylate and M2Mφ transfusion), GOX+M1(I) (treated by glyoxylate and M1Mφ induction), and GOX+M2(I) (treated by glyoxylate and M2Mφ induction). Each glyoxylate treatment was performed as a 6-d intra-abdominal injection of 80 mg kg^−1^ glyoxylate as described previously^52^.

Blood, 24-h urine samples, and kidney tissues were obtained from six mice from each group at d 6. Blood and urinary biochemistry was examined by Mitsubishi Chemical Medicine (Tokyo, Japan). Urinary pH and volumes were measured manually. Urinary oxalate concentrations were analyzed using FOM-110A (Hokuto Denko Co., Tokyo, Japan)^53^. The urine was centrifuged at 1500 × *g* for 15 min, and 100 μl sediment samples were observed at 400 × magnification using the AX80 light optical microphotograph (Olympus, Tokyo, Japan) to detect and quantify the number of CaOx crystals.

### Transfusion of *in vitro*-derived M1Mφs and M2Mφs

After the culture and differentiation of M1Mφs and M2Mφs from BMMs, 1.0 × 10^6^ viable Mφs were separated with the medium. Mice were anesthetized by sevoflurane and transfused with either M1Mφs or M2Mφs via a single tail-vein injection using a 27 gauge needle at 1 d prior to glyoxylate administration as described previously^23^.

### Induction of renal M1Mφs and M2Mφs in experimental mice

Daily intra-abdominal injections of 50 μg body^−1^ LPS (Sigma-Aldrich) and 1.0 μg body^−1^ IFN-γ (BioLegend, San Diego, CA), 5.0 μg body^−1^ IL-4 (BioLegend), and 5.0 μg body^−1^ IL-13 (BioLegend) were performed concurrently with glyoxylate treatment in order to induce the migration of M1Mφs and M2Mφs^12^,^54^,^55^,^56^,^57^,^58^.

### Flow cytometry of renal mononuclear cells

We prepared enriched CD11b^+^ and/or CD11c^+^ cells as single-cell suspensions using mouse CD11b- or CD11c-microbeads and auto-MACS (Miltenyi Biotec), following the manufacturer’s protocol. Next, CD11b and/or CD11c cells from mouse kidneys and BMM-derived M1/M2Mφs were stained with Ly-6C CD45, CD11b, CD11c, CD163, CD206, and F4/80 of their antibodies (Supplementary Table S2) and harvested using FACS Canto II (Becton Dickinson, San Jose, CA). Data were analyzed using FlowJo 10 (Tree Star, Palo Alto, CA).

### Detection of kidney CaOx crystals

Crystal formation in the extracted mouse kidneys was examined using Pizzolato staining, as described previously^59^. Non-stained sections were observed using the AX80 polarized light optical microphotograph (Olympus). Crystal formation was calculated as the percent area of CaOx crystal deposition per kidney section and assessed quantitatively using Image Pro Plus (Media Cybernetics, Inc., Bethesda, MD).

### Immunohistochemical staining

OPN, CD44, MCP-1, FN, and Vcam1 were immunohistochemically stained on 4-μm-thick cross-sections treated with microwave irradiation for 15 min and blocked with 0.5% H_2_O_2_ in methanol for 30 min, followed by washing in 0.01 M PBS, and further treated with skimmed milk in PBS for 1 h at room temperature. These slides were incubated in primary antibodies overnight at 4 °C, and the reacted antibody was then detected using secondary antibodies^7^,^28^. Antibodies are shown in Supplementary Table S3.

### RNA extraction and qRT-PCR

Total RNA was extracted and reverse transcribed into cDNA. Then, qRT-PCR was performed with TaqMan Universal PCR Master Mix (404437; Applied Biosystems) using the 7500 FAST Real-time PCR System (Applied Biosystems). After denaturing at 95 °C for 10 min, PCR cycling was performed with each cycle consisting of 95 °C for 15 s followed by 60 °C for 1 min. The PCR reaction was repeated 40 times. cDNA amplification was compared with that of control samples, and the expression ratios were determined using a standard curve prepared from a 35-dilution series of control samples and corrected for the amount of total RNA^23^. TaqMan Probes are shown in Supplementary Table S4. The expression of each gene was normalized to that of β-actin. The corrected expression of each gene was normalized to the average value of the control group for the *in vivo* study and of the RTC or BMM without COM exposure group for the *in vitro* study.

### ELISA

ELISAs were performed to measure the levels of soluble OPN (Mouse Osteopontin Assay Kit, IBL, Gunma, Japan), MCP-1 (Mouse CCL2/JE/MCP-1 Immunoassay, R&D Systems, Minneapolis, MN), IL-6 (Mouse IL-6 Immunoassay, R&D Systems), and TNFα (Mouse TNFα Immunoassay, R&D Systems) produced in the supernatant of each culture dish according to the manufacturer’s instructions.

### Collection of human renal papillary tissues

The genome-wide analysis of human papillary tissue was approved by the Nagoya City University Ethics Board (No. 929), and these protocols were conducted in accordance with the Declaration of Helsinki. All participants provided informed consent prior to the surgery. We obtained renal papillary tissue biopsies of six idiopathic CaOx stones from participants who underwent retrograde intrarenal surgery at our institutions in November 2013–April 2015. Patients with active urinary tract infection, metabolic and autoimmune disease, carcinoma, and severe hydronephrosis (Grade 3 or 4, according to The Society for Fetal Urology guidelines) were excluded. In each patient, samples were individually collected from the renal papillary tissue with RP (Plaque group) and the normal papillary tissue without RP (Normal group). The control group consisted of normal renal papillary tissues collected from six patients who underwent ureteroscopy or nephrectomy either for hemorrhage screening or for adrenal tumor adhesion without urolithiasis.

### Microarray analysis

Total RNA was extracted from the human renal papillary tissues in RNAlater^®^ using the RNeasy Micro Kit (Qiagen). cDNAs amplified using the Ovation Pico System (Nugen, San Carlos, CA) were subjected to transcriptome analysis using Agilent SurePrint G3 microarrays. Microarray data were analyzed using GeneSpring 13.1 (Agilent Technologies, Santa Clara, CA). Changes in gene expression greater than 2-fold between groups were considered significant (*p* < 0.01). All microarray data were deposited in the Gene Expression Omnibus (Acc. No: GSE 73680).

### Statistical analysis

All data are expressed as mean ± standard deviation. Two-way analysis of variance was performed for identifying differences among three or more groups or the Mann-Whitney *U* test for identifying differences between two groups. Categorical data were compared using Fisher’s exact test. Differences were considered statistically significant at *p* < 0.05. All statistical analyses were performed using SAS 9.1 (SAS Institute Inc., Cary, NC).

## Additional Information

**How to cite this article**: Taguchi, K. *et al.* M1/M2-macrophage phenotypes regulate renal calcium oxalate crystal development. *Sci. Rep.* **6**, 35167; doi: 10.1038/srep35167 (2016).

## Supplementary Material

Supplementary Information

## Figures and Tables

**Table 1:** Serum and urinary variables in the control, glyoxylate (GOX)-treated, Mφ-transfused, and Mφ-induced groups.

*Serum*	Control	GOX	Mφ - transfusion		Mφ - induction	
			GOX+M1(T)	GOX+M2(T)	GOX+M1(I)	GOX+M2(I)
Cr (mg/dl)	0.09 ± 0.02	0.15 ± 0.01	0.16 ± 0.02	0.14 ± 0.01	0.27 ± 0.05^#,§,^*	0.09 ± 0.01^§,^*
Ca (mg/dl)	8.68 ± 0.34	8.69 ± 0.15	8.20 ± 0.15	8.69 ± 0.13	7.57 ± 0.35	8.16 ± 0.30
P (mg/dl)	5.92 ± 0.46	6.49 ± 0.26	5.25 ± 0.38	6.66 ± 0.29	9.93 ± 1.01^#,§,^*	6.54 ± 0.33*
Mg (mg/dl)	3.16 ± 0.28	3.29 ± 0.16	3.23 ± 0.17	2.84 ± 0.04	3.70 ± 0.52	3.23 ± 0.16
Na (mg/dl)	143.2 ± 1.2	146.5 ± 1.0	148.0 ± 0.9	146.0 ± 1.1	151.3 ± 3.3^#^	151.7 ± 1.0^#,§^
K (mg/dl)	7.42 ± 0.56	6.55 ± 0.39	6.68 ± 0.20	6.57 ± 0.18	7.47 ± 0.69	6.86 ± 0.39
Volume (ml)	0.60 ± 0.23	1.77 ± 0.22^#^	1.35 ± 0.25	1.11 ± 0.18	0.80 ± 0.51*	2.27 ± 0.30^#,^*
pH	6.30 ± 0.20	6.70 ± 0.28	7.13 ± 0.72	7.29 ± 0.47	6.20 ± 0.18	6.08 ± 0.08
Ca (μmol · g^−1^ · Cr^−1^)	4.71 ± 1.58	3.09 ± 0.35	2.56 ± 1.28	1.92 ± 0.29	2.61 ± 0.95	2.08 ± 0.29
P (μmol · g^−1^ · Cr^−1^)	148 ± 43	152 ± 27	167 ± 39	151 ± 32	212 ± 76	186 ± 38
Mg (μmol · g^−1^ · Cr^−1^)	32.5 ± 3.3	45.9 ± 10.4	40.1 ± 22.9	35.5 ± 13.5	55.8 ± 16.3	62.1 ± 7.2
Na (μmol · g^−1^ · Cr^−1^)	132 ± 12	161 ± 12	127 ± 26	151 ± 13	175 ± 33	230 ± 21^#^
K (μmol · g^−1^ · Cr^−1^)	125 ± 17	159 ± 8	131 ± 34	137 ± 8	180 ± 19	158 ± 12
Ox (μmol · g^−1^ · Cr^−1^)	50 ± 5	112 ± 13^#^	137 ± 14^#^	115 ± 11^#^	122 ± 11^#^	114 ± 12^#^
Cit (μmol · g^−1^ · Cr^-1^)	423 ± 146	613 ± 117	620 ± 114	401 ± 74	403 ± 105	592 ± 46

Mean ± standard error (SE). ^#^Indicates significant differences compared with the control group at *p* < 0.05; ^§^indicates significant differences compared with the glyoxylate (GOX)-treated group at *p* < 0.05; *indicates significant differences between the M1 and M2 groups of each transfusion or induction type at *p* < 0.05. Abbreviations: Cr, creatinine; Ca, calcium; P, phosphorus; Mg, magnesium; Na, sodium; K, potassium; Ox, oxalate; Cit, citrate.
