# Supplementary material for: M1/M2-macrophage phenotypes regulate renal calcium oxalate crystal development
Source: Sci Rep. 2016 Oct 12;6:35167. doi: 10.1038/srep35167 (PMC5059697; doi:10.1038/srep35167)

SUPPLEMENTARY INFORMATION

Title:

M1/M2-macrophage phenotypes regulate renal calcium oxalate crystal development

Authors:

Kazumi Taguchi, Atsushi Okada*, Shuzo Hamamoto, Rei Unno, Yoshinobu Moritoki, Ryosuke Ando, Kentaro Mizuno, Keiichi Tozawa, Kenjiro Kohri, Takahiro Yasui

Department of Nephro-urology, Nagoya City University Graduate School of Medical Sciences, Nagoya, Japan.

Supplementary Table 1. *Information on the age, gender, side of tissue collection, basic metabolic index (BMI), and serum and urinary variables of patients*

		Control (n = 6)	CaOx stone former (n = 6)	P value
General				
Age	y.o	49 ± 7	56 ± 4	N.S*
Gender	M, F	3, 3	4, 2	N.S#
Side	Rt, Lt	3, 3	2, 4	N.S#
BMI	kg/m^2	22.7 ± 1.1	22.3 ± 1.8	N.S*
Serum				
BUN	mg/dl	13.2 ± 1.6	14.2 ± 1.8	N.S*
Cre	mg/dl	0.9 ± 0.2	0.8 ± 0.1	N.S*
Ca	mg/dl	9.4 ± 0.3	9.5 ± 0.2	N.S*
P	mg/dl	2.9 ± 0.5	3.1 ± 0.2	N.S*
UA	mg/dl	5.1 ± 0.5	5.8 ± 0.3	N.S*
WBC	cells/μl	6650 ± 765	7200 ± 875	N.S*
CRP	mg/dl	2.1 ± 1.8	0.1 ± 0.1	N.S*
Urine				
pH		6.8 ± 0.6	6.6 ± 0.7	N.S*
WBC	cells/HPF	4 ± 2	26 ± 16	N.S*
RBC	cells/HPF	19 ± 16	37 ± 14	N.S*

Mean ± standard deviation (SD). * Mann-Whitney *U* test, § Student's t-test, and # Fisher's exact test.

Abbreviations: CaOx, calcium oxalate; BUN, blood urea nitrogen; Cre, creatinine; Ca, calcium; P, phosphorus; UA, uric acid; WBC, white blood cell; CRP, c-reactive protein; RBC, red blood cell; y.o, years old; M, male; F, female; Rt, right; Lt, left; HPF, high power field, NS, not significant.

Supplementary Table 2. Primary/secoundary antibodies for flow cytometry.

Protein Name	Antibodies	Product Codes
Primary		
Ly-6C	V450 rat anti-mouse Ly-6C (AL-21)	560894 (BD Horizon)
CD45	Brilliant Violet 510 anti-mouse CD45 (30-F 11)	103137 (BioLegend)
CD11b	FITC rat anti-mouse CD11b (M1/70)	555310 (BD Pharmingen)
CD11c	PE hamster anti-mouse CD11c (HL3)	553802 (BD Pharmingen)
CD163	rabbit anti-mouse CD163 polyclonal IgG (M-96)	sc33560 (Santa Cruz Biotechnology)
CD206	APC anti-mouse CD206 (MMR) (C068C2)	141707 (BioLegend)
F4/80	APC/Cy7 anti-mouse F4/80 (BM8)	123117 (BioLegend)
Secoundary		
	goat anti-rabbit IgG, F(ab')2-PE-Cy7	sc3845 (Santa Cruz Biotechnology)

Supplementary Table 3. Primary antibodies for immunohistochemical stain.

Protein Name	Antibodies	Product Codes
OPN	anti-mouse OPN (O-17) rabbit polyclonal antibody	18621 (IBL Co., Ltd.)
CD44	anti-mouse CD44 (IM7.8.1) rat monoclonal antibody	822201 (BioLegend)
MCP-1	anti-mouse MCP-1 (ab9669) rabbit polyclonal antibody	ab9669 (Abcam)
FN	anti-mouse FN (H-300) rabbit monoclonal antibody	sc9068 (Santa Cruz
VCAM1	anti-mouse VCAM1 (EPR5047) rabbit polyclonal antib	ab134047 (Abcam)

Supplementary Table 4. TaqMan primer information which were used for Quantitative Reverse Transcription polymerase chain reaction.

Probe	Gene Name	TaqMan Assay ID
Spp1	secreted phosphoprotein 1	Mm00436767_m1
Cd44	cd44	Mm01277163_m1
Ccl2	chemokine (CC motif) ligand 2	Mm00441242_m1
C3	complement component 3	Mm01232779_m1
Fn1	fibronectin 1	Mm01256744_m1
Vcam1	vascular cell adhesion molecule 1	Mm01320970_m1
Il6	interleukin 6	Mm00446190_m1
Nos2	nitric oxide synthase 2	Mm00440502_m1
Tnf	tumor necrosis factor	Mm00443258_m1
Arg1	arginase	Mm00475988_m1
Chi3l3	chitinase 3-like 3	Mm04213363_u1
Il10	interleukin 10	Mm00439614_m1
Pparg	peroxisome proliferator activated receptor gamma	Mm00440940_m1
Retnla	resistin like alpha	Mm00445109_m1

Supplementary Figure 1. The expression of each protein contained in each Mφ culture medium. The expression of each protein that was secreted in the supernatant was determined by Enzyme-linked immunoSorbent Assay (ELISA). Data are presented as means ± standard errors. N = 6 for each group. * indicates significant differences among three different macrophages at $p < 0.05$; # indicates significant differences in the mono-culture of each macrophage group at $p < 0.05$; § indicates significant differences in the mono-culture of each macrophage group incubated with COM crystals at $p < 0.05$. OPN, osteopontin; MCP-1, monocyte chemoattractant protein-1; IL-6, interleukin-6; TNFα, tumor necrosis factor α; RTC, renal tubular cell.

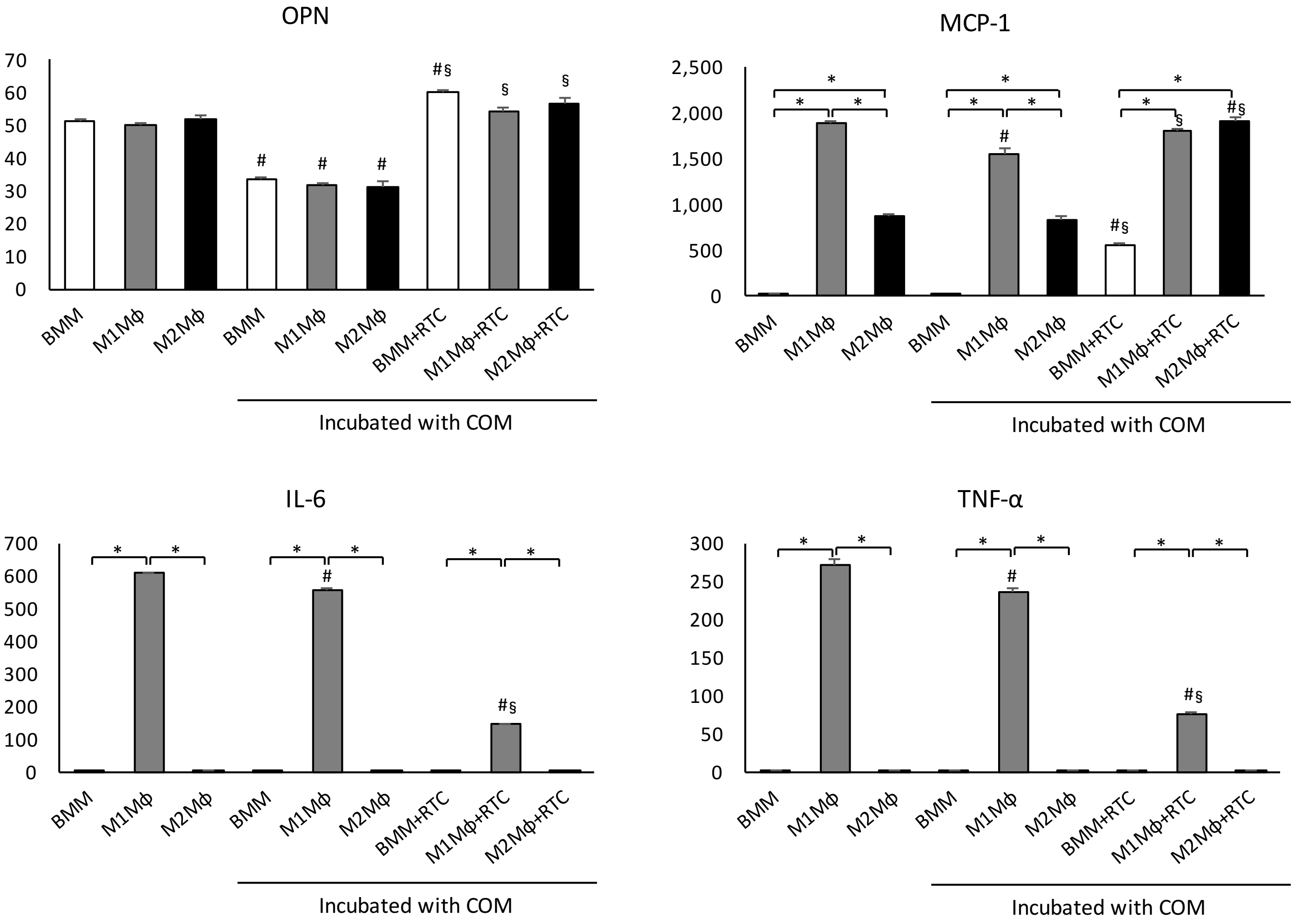

Supplementary Figure 2. The ratio of renal crystal deposits/ urinary crystal excretion. Either ratio of renal crystal deposits or urinary crystal excretion were calculated using the ratio of the GOX group as average, which were described in Figure 3B and 3C.

N = 6 for each group. Data are presented as means ± standard errors. * indicates significant differences compared with the glyoxylate (GOX)-treated group. M1 (T) and M2 (T) indicate transfusion, whereas M1 (I) and M2 (I) induction treatment of each macrophage.

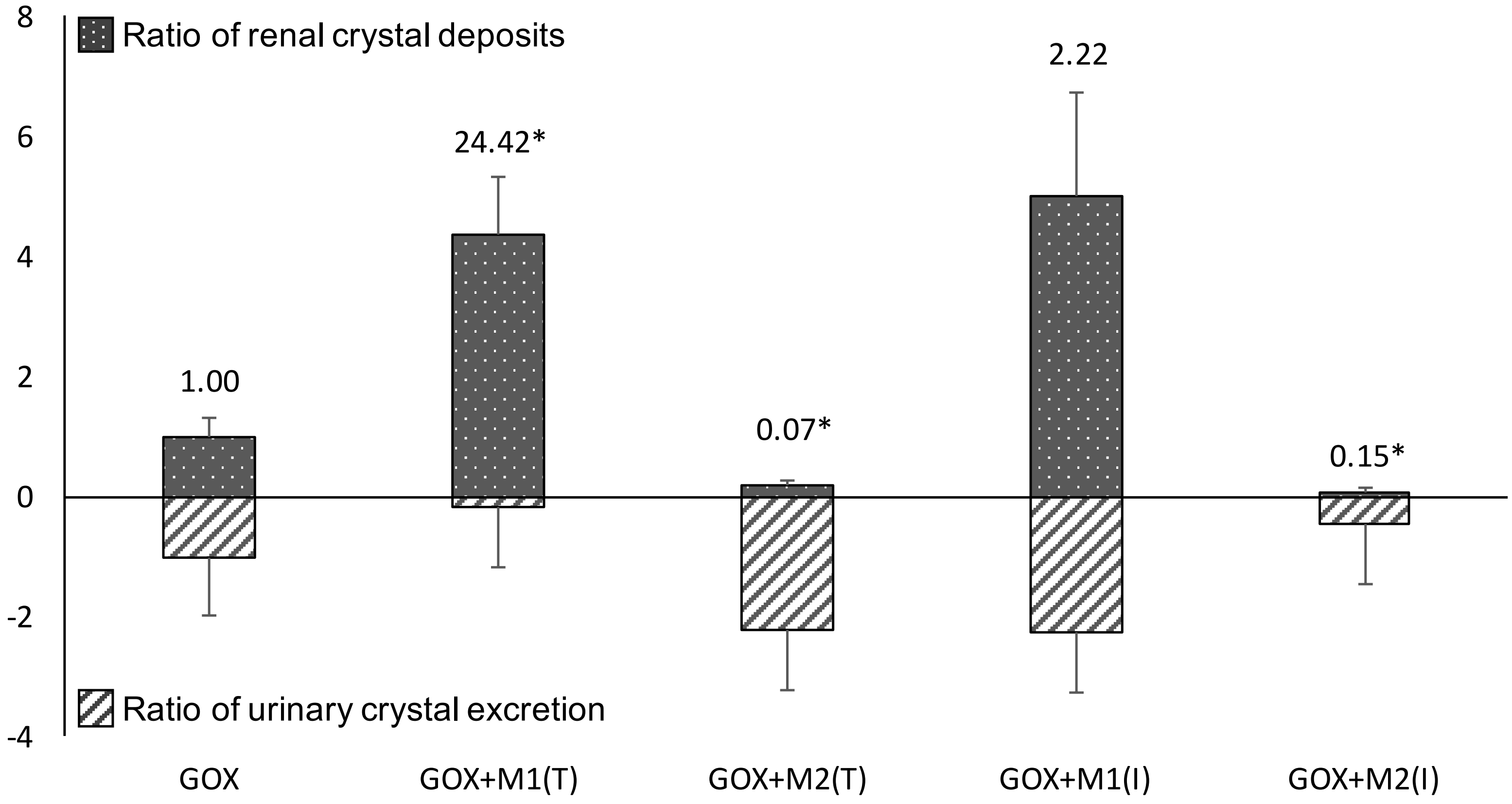

Supplement: Supplementary Information [file srep35167-s1.pdf]
